# Iron nanoparticle/carbon nanotube composite as oxidase-like nanozyme for visual analysis of total antioxidant capacity

**DOI:** 10.1016/j.fochx.2024.102093

**Published:** 2024-12-12

**Authors:** Junlin Liu, Sophia Xie, Nan Wang, Zhongyue Sun, Lina Tang, Guo-jun Zhang, John Tressel, Yulin Zhang, Yujie Sun, Shaowei Chen

**Affiliations:** a School of Laboratory Medicine, Hubei University of Chinese Medicine, Hubei Shizhen Laboratory, Wuhan 430065, China; b Wuhan Britain-China School, Wuhan 430033, China; c Department of Physics, Jinan University, Guangzhou 510632, China; d Department of Chemistry and Biochemistry, University of California, Santa Cruz, CA 95060, USA; e Hubei Key Laboratory of Resources and Chemistry of Chinese Medicine, School of Pharmacy, Hubei University of Chinese Medicine, Wuhan 430065, China

**Keywords:** Carbon nanotube, Iron nanoparticle, Oxidase, Nanozyme, Total antioxidant capacity, Fluorescence, Smartphone

## Abstract

Total antioxidant capacity (TAC) is an important indicator for assessing the merit of natural plants and foods. Herein, a visual TAC assay is developed based on the oxidase-like activity of nitrogen-doped carbon nanotubes loaded with Fe nanoparticles (FeNPs@NCNT), which is prepared via high-temperature pyrolysis of metal-organic framework precursors and can catalyze the oxidation of colorless *o*-phenylenediamine (OPD) to colored 2,3-diaminophenazine (DAP). The addition of antioxidants (e.g., quercetin) impedes the formation of DAP, diminishing the color change, which can be analyzed via the RGB values obtained with a smartphone color-recognition APP, “Color Picker”. The change of the optical signal can also be analyzed in the fluorescence mode. These two detection modes yield consistent TAC analysis of actual plant samples, in accord with results from the standard ABTS method. Results from this study highlight the unique potential of nanozymes in the development of effective TAC analysis platforms for natural plants and food.

## Highlights

• Carbon nanotubes loaded with iron nanoparticles by pyrolysis of MOF precursors.
• FeNPs@NCNT composites as POD and OXD mimics.
• Colorimetric assay of total antioxidant capacity by fluorescence and smartphone APP.
• Detection of total antioxidant capacity of actual plants and foods.

## 1 Introduction

Excessive free radicals and oxidants in the body can lead to many diseases and rapid aging (Di Meo & Venditti, 2020). Active antioxidant ingredients in many foods and medicinal plants effectively scavenge excess free radicals and oxidants, thereby exerting their anti-inflammatory, anti-aging and immune-boosting effects (J. R. Chen et al., 2023; Fu et al., 2022; Jiang et al., 2018; X. S. Li et al., 2020; Y. Wang et al., 2023). Therefore, analyzing the total antioxidant capacity (TAC) is of great significance for disease diagnosis, pathogenesis studies, and food and drug quality assessments (Zhen et al., 2016). This is typically based on colorimetric, electrochemical, fluorescent, chromatographic, chemiluminescent and mass spectrometry methods (Iranifam & Al Lawati, 2019; P. J. Ni et al., 2021; Shi et al., 2022; Tabart, Kevers, Pincemail, Defraigne, & Dommes, 2010; Yalçin et al., 2021; Ziyatdinova, Kozlova, & Budnikov, 2016). Among these, visual assays stand out for on-site detection of TAC due to simple and cost-effective operation and no requirement of complex instrumentation. In fact, development of low-cost, convenient and effective visual methods for on-site detection of TAC has been attracting a great deal of interest lately.

Nanozymes are a class of nanomaterials with catalytic activities similar to those of natural enzymes, and have been used extensively in various fields, such as disease diagnosis and treatment, environmental treatment, and industrial production, due to their superb stability, good environmental tolerance, diversity of enzyme activities and low production cost (Ren et al., 2022; Zandieh & Liu, 2024). Among these, peroxidase (POD)-like nanozymes can catalyze the decomposition of H_2_O_2_ to hydroxyl radicals (•OH), which can then oxidize the colorless *o*-phenylenediamine (OPD) to colored 2,3-diaminophenazine (DAP) (M. L. Chen et al., 2024). The addition of active antioxidant ingredients can deplete the hydroxyl radicals, leading to a diminishment of the production of the colored DAP. Thus, POD nanozymes can be used for the visual analysis of TAC. For instance, Zhang et al. synthesized nitrogen (N), phosphorus (P), and sulfur (S) codoped carbon nanozyme (NPS-C) via one-step high-temperature pyrolysis and observed a high POD-like activity (Y. S. Wang et al., 2023). The N and P dopants acted as electron donors and enhanced the adsorption of the reactants, whereas the S dopants optimized the geometry of the carbon structure and acted as electron acceptors to facilitate electron transfer during the reaction. The doping of these nonmetal elements altered the electron distribution within the carbon scaffold, facilitated electron transfer between the nanozymes and reactants, and optimized the pathway for catalytic oxidation, thus enhancing the POD-like enzyme activity. A colorimetric biosensor based on the NPS-C nanozymes achieved successful accurate assessment of TAC in commercial beverages and demonstrated potential applications in food safety. Ni et al. constructed a colorimetric sensing platform based on copper selenide nanoparticles (Cu_2-x_Se NPs) that featured a POD-like enzyme activity. The copper-based nanozyme exhibited a Fenton-like activity, where the unique copper vacancy structure (coexistence of Cu^2+^ and Cu^+^) of Cu_2-x_Se provided an effective charge transfer pathway. In fact, the nanozyme effectively catalyzed the oxidation of colorless 3,3′5,5′-tetramethylbenzidine (TMB) to blue oxidized TMB (oxTMB), which was successfully exploited for the visual detection of TAC (H. H. Ni, Li, Yan, Huang, & Zou, 2024). However, these POD nanozyme based methods require the addition of unstable H_2_O_2_，which makes the procedure cumbersome and compromises the accuracy of the analysis results. By contrast, nanozymes with oxidase (OXD)-like activity can directly catalyze the reduction of O_2_ to superoxide radical anions ($O_{2}^{∙-}$) which then oxidize the colorless OPD to colored DAP (Chong, Liu, & Ge, 2021). In the presence of active antioxidant ingredients, $O_{2}^{∙-}$ can be readily depleted, which inhibits the production of colored DAP. Thus, OXD nanozyme-based assays render it possible to visualize TAC analysis without the addition of H_2_O_2_.

The OXD-like activity is the most critical factor in determining the performance of visual analysis of TAC. A range of noble metal-based nanomaterials (e.g., Au, Ag, Pt, and Pd) exhibit good OXD-like activity and achieve desirable results in TAC analysis. For instance, Xu et al. synthesized a nanozyme based on polyvinylpyrrolidone-capped gold nanoparticles (AuNPs-PVP) and observed an excellent OXD-like activity in catalyzing the oxidation of colorless TMB to blue oxTMB, which mitigated the issue caused by the instability of H_2_O_2_. Meanwhile, as the nanozyme could act as an active substrate for surface-enhanced resonance Raman scattering (SERRS), a strong SERRS signal was generated when TMB was oxidized to oxTMB. The signal was diminished with the addition of antioxidants (e.g., ascorbic acid). Both the attenuation of the signal and the diminution of the color could be exploited for TAC analysis (Xu, Song, & Xia, 2024). Similarly, Wei et al. prepared a platinum‑nickel nanoparticle-based nanozymes (Pt—Ni NPs) and observed a high OXD-like activity, as manifested again in the oxidation of TMB to oxTMB, and the color of oxTMB became lighter upon the addition of active antioxidant substances. Such a Pt—Ni NPs-based colorimetric platform was used for the successful detection of the antioxidant levels of small bioactive molecules, two antioxidant nanomaterials and three cells (X. Y. Wang et al., 2023). Yet, the low natural abundance of precious metals and their high costs have limited their wide-spread applications in TAC analysis.

Thus, extensive research has been devoted to the development of effective assays based on earth-abundant and low-cost transition metals, such as Fe, Co, and Ni (J. Q. Li, Mao, Zhang, Wang, & Feng, 2023; Liu et al., 2024; Zhang et al., 2024). The empty d or f orbitals of transition metals make it energetically favorable to form coordination bonds with substrate molecules, lowering the energy barrier of the formation of transition states and facilitating the catalytic process (Kong, Liang, Wang, & Wu, 2023). Among them, Fe-based nanozymes have indeed been used for TAC detection (X. Q. Wang et al., 2023). However, most of the Fe-based nanozymes exhibit POD rather than OXD activity (Jiao et al., 2020). Therefore, synthesis of Fe-based nanomaterials with high OXD-like activity is of great significance but remains challenging.

Carbon nanotubes (CNTs) possess a high specific surface area, excellent electrical conductivity and good thermochemical stability, and can be used as a unique structural scaffold for loading various types of nanoparticles. In addition, heteroatom doping (e.g., N doping) can modulate the sp^2^ hybridized carbon matrix and improve the performance of CNTs (Dubey, Dutta, Sarkar, & Chattopadhyay, 2021). Moreover, nitrogen dopants can form coordination with the metals in the nanoparticles, which is conducive to stabilizing the nanoparticles. This can lead to enhancement of the OXD activity and durability.

**Scheme 1:**
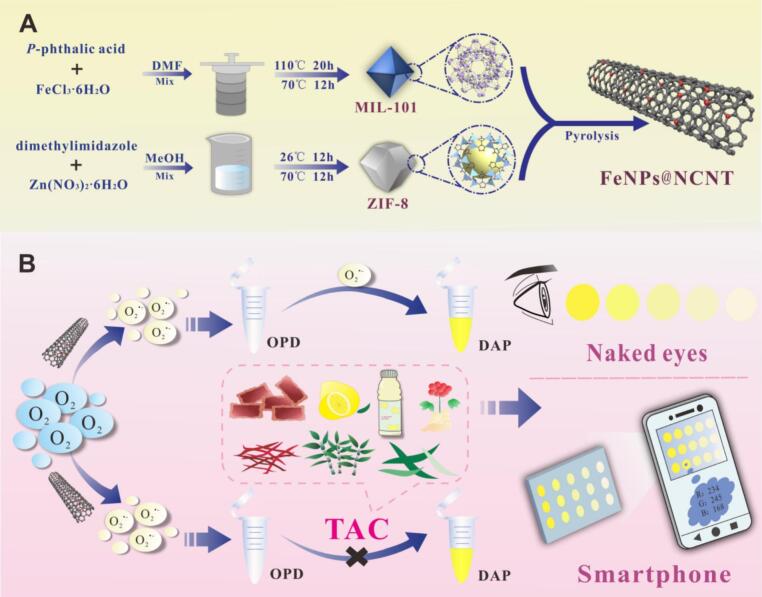
(A) Schematic diagram of the synthesis of FeNPs@NCNT. (B) Principle of the FeNPs@NCNT-based method for the detection of total antioxidant capacity in real samples.

Herein, nitrogen-doped carbon nanotubes loaded with Fe nanoparticles (FeNPs@NCNT, Scheme 1A) were prepared by pyrolysis of two metal organic framework (MOF) precursors, zeolitic imidazolate framework-8 (ZIF-8) and iron-containing MIL-101. In the obtained samples, Fe NPs were uniformly distributed in the CNT skeletons as the main active sites, and the N dopants exhibited a synergistic effect on the electronic structure and chemical properties of the CNTs leading to enhanced catalytic activity. Notably, the FeNPs@NCNT nanocomposites exhibited excellent OXD activity in the reduction of O_2_ to $O_{2}^{∙-}$, which then oxidized colorless OPD to yellow DAP. The visual signal was markedly weakened upon the introduction of active antioxidant ingredients like quercetin. Experimentally, the changes of the visual signals could be read, analyzed, and output even with a smartphone, a unique feature for rapid and accurate quantitative TAC detection (Scheme 1B). This can be exploited for the sensitive on-site analysis of TAC in resource-limited areas.

## 2 Experimental section

### 2.1 Reagents

Ferric chloride hexahydrate (FeCl_3_∙6H_2_O), *p*-phthalic acid (Pubchem CID:7489), dimethylformamide (DMF), zinc nitrate hexahydrate (Zn(NO_3_)_2_∙6H_2_O), dimethylimidazole, methanol (MeOH, Pubchem CID:7489), ethanol (EtOH, Pubchem CID:702), and 5,5-dimethyl-1-pyrroline N-oxide (DMPO, Pubchem CID:1774) were purchased from Aladdin (Shanghai, China). *O*-phenylenediamine (OPD, Pubchem CID:7243), hydrogen peroxide (H_2_O_2_, Pubchem CID:784), magnesium chloride (MgCl_2_, Pubchem CID:5360315), sodium nitrite (NaNO_2_, Pubchem CID:23668193), tryptophan (Pubchem CID:6305), and glucose (Glu, Pubchem CID:5793) were purchased from Sinopharm Chemical Reagents Co. ABTS reagent was purchased from Biyuntian Biotechnology Co. Bovine serum albumin (BSA) was purchased from Beijing Boao Toda Technology Co. All chemicals in this study were used directly without further purification. Ultrapure water was provided with a Millipore Milli-Q Direct8 water purification system (resistivity 18.2 MΩ cm).

### 2.2 Preparation of FeNPs@NCNT

The FeNPs@NCNT nanocomposite was prepared by adopting a procedure reported previously with slight modifications (M. Li et al., 2022), as shown in Scheme 1a. First, 2.56 g of FeCl_3_^.^6H_2_O and 0.61 g of *p*-phthalic acid were added into 45 mL of DMF under stirring, and then heated in a hydrothermal reactor at 110 °C for 20 h, producing MIL-101 after washing and drying overnight. Second, 2.52 g of Zn(NO_3_)_2_^.^6H_2_O was added into 120 mL MeOH along with 6 g of dimethylimidazole under magnetic stirring. ZIF-8 was obtained after centrifugation and rinsing followed by drying overnight. The two MOFs obtained above, MIL-101 and ZIF-8, were mixed and ball milled at a mass ratio of 1:5 under the protection of a N_2_ atmosphere, before being heated at 900 °C for 2 h in a N_2_ atmosphere to produce nitrogen-doped carbon nanotubes loaded with Fe nanoparticles (FeNPs@NCNT). A second heating treatment was carried out at 950 °C in a NH_3_ atmosphere to increase N doping.

### 2.3 Characterization

The morphology and structure of the obtained samples were investigated by scanning electron microscopy (SEM, Hitachi S-4800) and transmission electron microscopy (TEM, FEl Talos F200S) measurements. Elemental analyses were carried out using a PH Quantera X-ray electron spectroscopy (XPS) instrument, where all binding energies were calibrated against the C 1s peak (284.8 eV). The crystallinity of the samples was assessed by X-ray diffraction measurements (XRD, X'Pert Pro Super). Electron paramagnetic resonance (EPR) tests were performed on an EMX micro-6/1/P/L instrument.

### 2.4 OXD and POD activities of FeNPs@NCNT

In OXD activity assessments, FeNPs@NCNT (6 μL, 1 mg mL^−1^) and OPD (6 μL, 1 mg mL^−1^) were added to a phosphate buffer solution (PBS, pH = 7.2), with the final total volume fixed at 200 μL. Then, the optical absorbance of the oxidation product (DAP) at 420 nm was recorded after 30 min's reaction at room temperature. The POD activity of FeNPs@NCNT was evaluated in the same manner but with the addition of H_2_O_2_ (30 μM). To detect the free radical species during the reaction, FeNPs@NCNT was dispersed into an aqueous solution containing H_2_O_2_ (2 μL, 30 mM) and DMPO (5 μL, 100 mM), or a methanol solution containing only DMPO (5 μL, 100 mM) for EPR measurements.

### 2.5 Kinetic study of FeNPs@NCNT

To study the steady-state kinetics of FeNPs@NCNT with the OPD substrate, OPD at different concentrations (i.e., 0.1, 0.2, 0.4, 0.6, 0.8, and 1.0 mM) was added into a 96-well plate containing FeNPs@NCNT (6 μL, 30 μg mL^−1^). The final volume of the reaction system was fixed at 200 μL and the absorbance of the reaction system at 420 nm was recorded. Non-linear regression analysis was performed based on the Michaelis-Menten equation, $v=\frac{v_{\max}\left[s\right]}{K_{m}+\left[s\right]}$, where *v* is the reaction rate, *v*_max_ the maximum reaction rate (at saturated substrate concentration), [S] the substrate (OPD) concentration, and K_m_ the Michaelis-Menten constant.

### 2.6 TAC analysis

Quercetin at different concentrations was added into a PBS (pH = 7.2) solution containing FeNPs@NCNT (30 μg mL^−1^) and OPD (30 μg mL^−1^). After 30 min's incubation at room temperature, digital images were acquired using the rear camera of a smartphone and the RGB values of the digital images were read through the “Color Picker” APP (details in **Fig. S1**). Meanwhile, the fluorescence signals were obtained at the excitation wavelength of 417 nm.

### 2.7 TAC analysis of actual samples

The practical application of the FeNPs@NCNT platform was evaluated via determining the TAC of eight actual samples that included *Carthami flos* (Sample 1), *Eucommia ulmoides* (Sample 2), *Dendrobii Caulis* (Sample 3), *Panax notoginseng* (Sample 4), *Panax ginseng* (Sample 5), *Camellia sinensis* (Sample 6), *Citrus limon* (Sample 7), and commercial beverage (Sample 8). Experimentally, dry *Carthami flos*, *Eucommia ulmoides*, *Dendrobii Caulis*, *Panax notoginseng*, *Panax ginseng* and *Camellia sinensis* were ground into powders; and 1 g of the powders was mixed with 40 mL of anhydrous ethanol under vortex for 5 min, and then sonicated at 60 °C for 120 min before being centrifuged at 10,000 rpm for 10 min. The supernatant was purified through a 0.22 μm filter and dried with a gentle nitrogen stream. The obtained concentrate was then added into a 1 mL mixed solution of PBS and EtOH (v:v = 1:1). For *Citrus limon*, the juice was collected from a juice blender and centrifuged three times at 10,000 rpm for 10 min each, before being purified through a 0.22 μm filter. Commercial beverages were filtered directly through a 0.22 μm filter. These samples were respectively added into the reaction system containing FeNPs@NCNT and OPD. After incubation for 30 min at room temperature, the optical changes were analyzed with a smartphone (Xiaomi 9 SE, Xiaomi Technology Co., Ltd., China) and fluorescence spectroscopy measurements. The standard procedure of TAC analysis (ABTS kit) was carried out to test the actual samples by following the kit operating instruction. The results obtained from these three methods (smartphone-based method, fluorescence-based method, and ABTS kit) were compared to examine their consistency.

## 3 Results and discussion

### 3.1 Structural characterization of FeNPs@NCNT

**Fig. 1:**
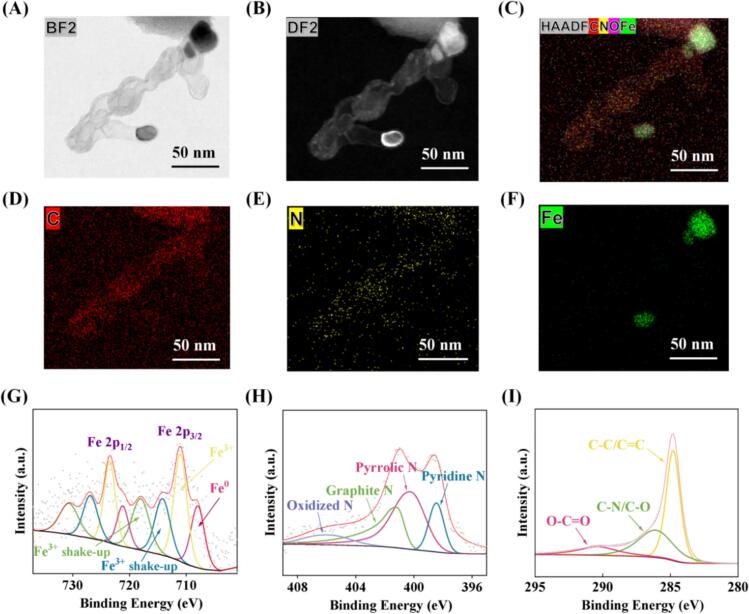
Characterization of FeNPs@NCNT. (A) Bright- and (B) dark-field TEM images of FeNPs@NCNT. (C—F) HAADF-STEM images and the corresponding elemental maps of FeNPs@NCNT. (G) High-resolution XPS scans of the (G) Fe 2p, (H) N 1s and (I) C 1s electrons of FeNPs@NCNT.

The materials structures were first characterized by SEM measurements. From **Fig. S2**, one can see that FeNPs@NCNT consisted of interwoven nanotubes that were decorated with a number of nanoparticles on the surface. Indeed, both bright-field (Fig. 1A) and dark-field (Fig. 1B) TEM measurements show a bamboo sheet-like structure, with dark nanoparticles encapsulated at the tips or edges of the nanotubes. Elemental mapping analyses based on energy-dispersive X-ray spectroscopy (EDS) (Fig. 1C–F and **Fig. S3**) show that the elements of C, N and O were distributed rather evenly across the nanotubes, whereas Fe was primarily confined within the dark-contrast nanoparticles. These results suggest the successful synthesis of carbon nanotubes loaded with Fe nanoparticles.

The elemental compositions and valency were then investigated by XPS measurements. From the survey spectrum in **Fig. S4A**, the C, N, O and Fe elements can be located at ca. 285, 400, 532 and 720 eV, respectively. The high-resolution scan of the Fe 2p electrons is shown in Fig. 1G, and the doublet at 711.0/723.4 eV can be ascribed to the 2p_3/2_/2p_1/2_ electrons of Fe(III), with the corresponding satellites at 714.2/726.8 and 718.0/730.7 eV. Metallic Fe can also be observed at 707.9/721.1 eV (Y. C. Wang et al., 2021). In the high-resolution scan of the N 1s electrons (Fig. 1H), four peaks can be deconvoluted at 398.4, 400.1, 401.2, and 405.8 eV, due respectively to pyridinic N, pyrrolic N, graphitic N, and oxidized N, confirming the successful doping of N into the carbon scaffold (Mercado et al., 2020). The C 1s and O 1s spectra are shown in Fig. 1I **and Fig. S4B**, respectively, which further confirmed the successful doping of N into the carbon skeleton and the absence of metal oxides.

XRD measurements (**Fig. S5**) show a strong diffraction peak at 2θ = 25.5° and several additional ones around 44°. The former can be ascribed to the (002) planes of graphitic carbon (PDF#75-1621), while the latter likely arose from the combined contributions of cubic Fe (110) and orthorhombic Fe_3_C (103), suggesting the formation of Fe/Fe_3_C nanoparticles in the nanocomposites (Jaiswal, Kumar, & Prakash, 2021), which is consistent with results from the above XPS measurements.

### 3.2 Enzyme-mimetic activities and mechanism of FeNPs@NCNT

**Fig. 2:**
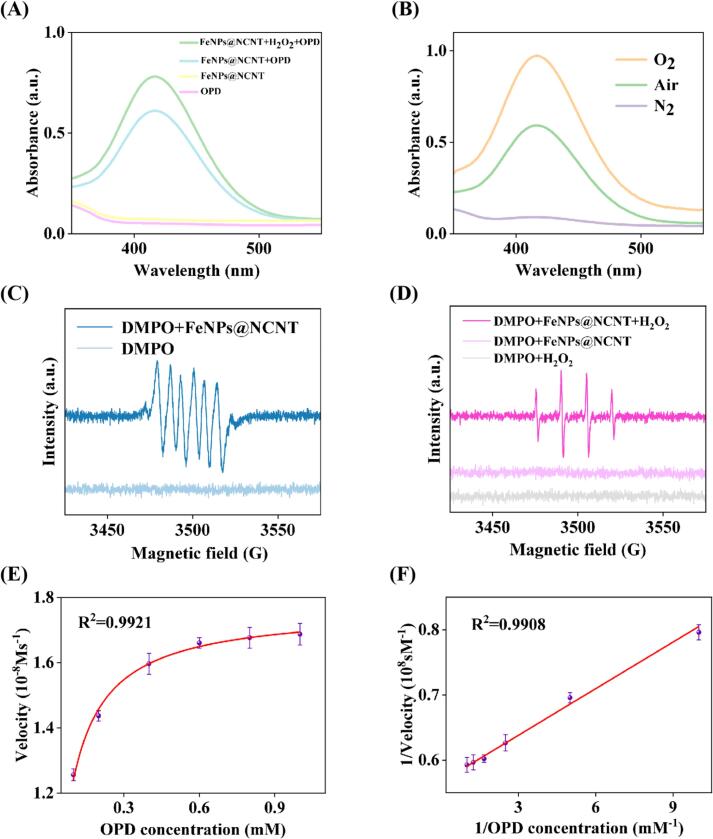
Enzymatic activity analysis of FeNPs@NCNT. (A) UV–vis absorption spectra of various solutions. (B) UV-vis absorption spectra of the FeNPs@NCNT+OPD solution when purged with O_2_, air or N_2_. (C—D) EPR spectra of various solutions in (C) methanol and (D) ultrapure water. (E) Steady-state kinetic assay and (F) Lineweaver-Burk plot for FeNPs@NCNT.

Notably, the obtained FeNPs@NCNT nanocomposite exhibited apparent OXD- and POD-like activities. From Fig. 2A, one can see that whereas OPD or FeNPs@NCNT alone showed only a featureless UV–vis absorption profile, the solution of FeNPs@NCNT and OPD exhibited a strong absorption peak at 420 nm, with an intense yellow color (**Fig. S6**). This suggests OXD-like activity of FeNPs@NCNT that oxidized the colorless OPD to colored DAP (Zhao et al., 2021). With the addition of H_2_O_2_, the solution showed an even more intense color and stronger absorption peak, indicating that FeNPs@NCNT also mimicked POD whereby H_2_O_2_ was catalytically decomposed to ·OH and facilitated the oxidation of OPD. These results showed that the FeNPs@NCNT nanocomposite possessed both OXD and POD activities. In fluorescence spectroscopy measurements, the colored solution can be seen to display an emission peak at 568 nm when excited at 417 nm, while no emission was observed with FeNPs@NCNT or OPD alone (**Fig. S7A**).

To explore the catalytic mechanism, the OXD-like activity of FeNPs@NCNT was investigated with the solution purged with O_2_, air, or N_2_. From Fig. 2B, one can see that the absorption peak intensity was markedly enhanced with the O_2_-saturated solution as compared to that with ambient air, whereas only a negligible absorption was observed with N_2_ purging. The fluorescence emission peak intensity showed a similar trend (**Fig. S7B**). These observations indicate that the formation of colored product catalyzed by FeNPs@NCNT strongly depended on oxygen dissolved in the solution.

In general, OXD-like nanozymes can effectively activate O_2_ to produce superoxide anions which then oxidize chromogenic substrates, such as OPD (M. Feng, Li, Zhang, & Huang, 2023). This was indeed confirmed by EPR measurements with DMPO as the spin trapping agent (Suzen, Gurer-Orhan, & Saso, 2017; Z. H. Wang, Ma, Chen, Ji, & Zhao, 2011). From the EPR spectra in methanol (Fig. 2C**)**, one can see that in the presence of FeNPs@NCNT, a sextet, with an intensity ratio of 1:1:1:1:1:1 (g = 2.0069), was observed within the magnetic field of 3450 to 3550 G, consistent with the patterns of DMPO-$O_{2}^{∙-}$ adducts (Yang et al., 2020), in sharp contrast to the featureless profile with the DMPO control. This implies that FeNPs@NCNT indeed catalyzed the reduction of O_2_ to $O_{2}^{∙-}$ which then oxidized OPD to DAP (Chang, Li, & Wu, 2024).

The POD mechanism of FeNPs@NCNT was also studied with DMPO as a spin trapping agent but in ultrapure water. As shown in Fig. 2D, in the FeNPs@NCNT + H_2_O_2_ solution, a quartet emerged within the magnetic field of 3450 and 3550 G, with an intensity ratio of 1:2:2:1 (g = 2.0058), due to the DMPO-·OH adducts (Ding et al., 2023), while no obvious signals were observed with FeNPs@NCNT or H_2_O_2_ alone, suggesting that FeNPs@NCNT catalyzed the decomposition of H_2_O_2_ to ·OH via POD-like activity, which then oxidized OPD to DAP.

These results confirmed that FeNPs@NCNT possessed both OXD and POD activities. This means that even in the absence of H_2_O_2_, FeNPs@NCNT could still catalyze the oxidation of the substrate. Therefore, to simplify the experimental procedure and avoid the issue of H_2_O_2_ instability, the subsequent studies were carried out by exploiting only the OXD activity of FeNPs@NCNT.

### 3.3 Kinetic analysis of FeNPs@NCNT

The steady-state kinetics of the OXD activity of FeNPs@NCNT was analyzed by varying the concentration of the OPD substrate. From the Michaelis-Menten curve (Fig. 2E) and Lineweaver-Burk plot (Fig. 2F**)**, the Mie constant (K_m_) and the maximum reaction velocity (*v*_max_) were quantitatively assessed after fitting the initial reaction velocity and OPD concentration. From **Table S1**, one can see that FeNPs@NCNT possessed a lower K_m_ (0.042 mM) and higher *v*_max_ (1.76 × 10^−8^ M s^−1^) for OPD, as compared to other types of nanozymes reported recently in the literature. This suggests that FeNPs@NCNT possessed a higher affinity for OPD and exhibited a superior OXD-like activity, most likely due to the enhanced affinity to oxygen species of the FeNPs@NCNT nanocomposites.

### 3.4 Optimization of the FeNPs@NCNT-based TAC performance

**Fig. 3:**
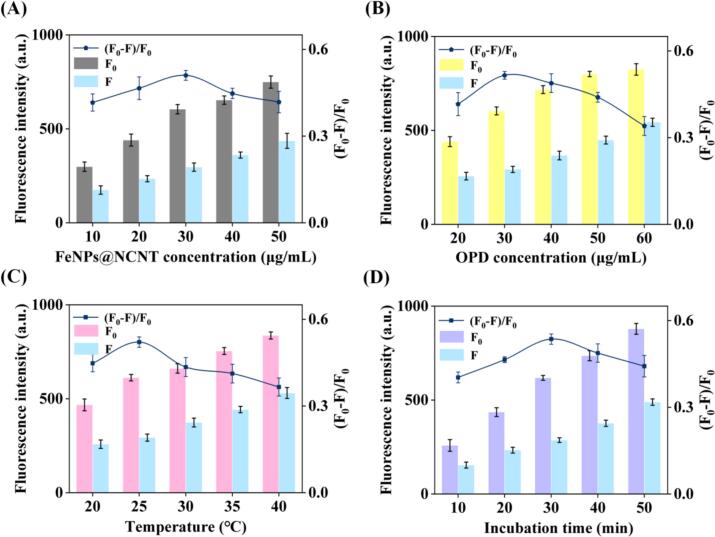
Optimization of reaction conditions for TAC analysis of quercetin (10 μg mL^−1^): (A) FeNPs@NCNT concentration (OPD concentration 30 μg mL^−1^; reaction temperature 25 °C; reaction time 30 min; pH = 7.2). (B) OPD concentration (FeNPs@NCNT concentration 30 μg mL^−1^_;_ reaction temperature 25 °C; reaction time 30 min; pH = 7.2) (C) Reaction temperature (FeNPs@NCNT concentration 30 μg mL^−1^_;_ OPD concentration 30 μg mL^−1^; reaction time 30 min; pH = 7.2). (D) Reaction time (FeNPs@NCNT concentration 30 μg mL^−1^_;_ OPD concentration 30 μg mL^−1^; reaction temperature 25 °C; pH = 7.2).

Notably, upon the addition of antioxidants like quercetin, the color appearance and the fluorescence emission intensity of the FeNPs@NCNT + OPD solution diminished markedly. Such a phenomenon can be exploited for TAC analysis. Yet the performance was found to be closely related to a number of factors, such as nanozyme concentration, OPD concentration, reaction time, temperature, and pH. To optimize the experimental conditions, the influence of these factors was investigated using the fluorescence response efficiency (FRE = (F_0_ − F)/F_0_, where F and F_0_ represent the fluorescence intensities in the presence and absence of quercetin, respectively) as an indicator. From Fig. 3A–B, one can see that both F_0_ and F increased with increasing concentration of the nanozyme and OPD. When both were at the concentration of 30 μg mL^−1^, FRE reached the maximum. Both F_0_ and F also increased with the increase of reaction temperature and time; and FRE reached the optimal value at 25 °C and 30 min (Fig. 3C–D). In addition, the FeNPs@NCNT-based platform exhibited the optimal performance at pH = 6.5 (**Fig. S8**), suggesting that a weakly acidic environment was favorable for the OXD activity, consistent with results from previous reports (K. Z. Feng et al., 2024). Nevertheless, one can see that an excellent performance was also observed in neutral media. Thus, to better align the TAC analysis with physiological environments, PBS (pH = 7.2) was used in subsequent experiments.

### 3.5 TAC performance of FeNPs@NCNT

**Fig. 4:**
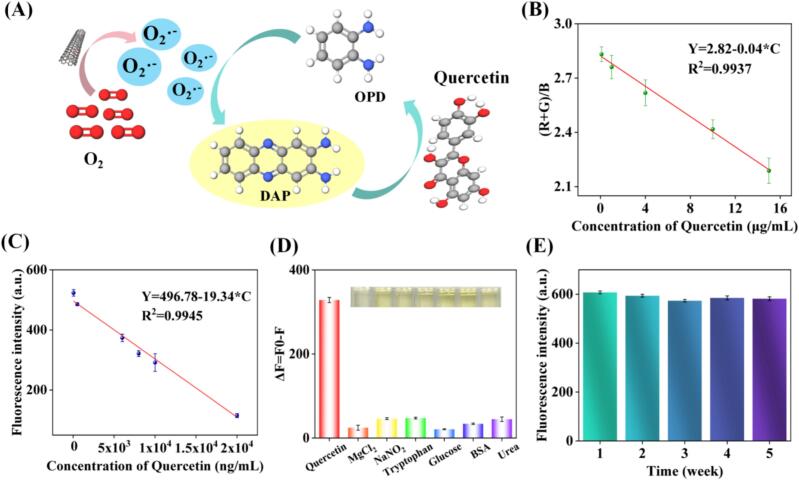
Performance of FeNPs@NCNT sensing platforms. (A) Mechanism of the reaction process. (B) Linear relationship between quercetin concentration and (R + G)/B value. (C) Linear relationship between quercetin concentration and fluorescence signal. (D) The selectivity of the TAC sensing platform. (E) The fluorescence signals of the blank by repeating the test for 5 consecutive weeks.

As demonstrated above, FeNPs@NCNT could directly catalyze the reduction of O_2_ to $O_{2}^{∙-}$, and induce the oxidation of colorless OPD to yellow DAP. Upon the addition of quercetin, $O_{2}^{∙-}$ was scavenged, thus inhibiting the oxidation of OPD, leading to a diminished color of the solution (Fig. 4A). Such a color change of the solution was clearly visible with naked eye and could be quantified via a smartphone-based digital image colorimetric analysis. After taking the photographs of different solutions with a smartphone, the color recognition APP (“Color Picker”) read the digital data of the three primary colors (RGB). As shown in **Fig. S9**, the color of the solutions gradually changed from bright yellow to colorless with the increase of quercetin concentration. Notably, there was a good linear relationship between (R + G)/B and quercetin concentration (C) within the range of 0.1–15 μg mL^−1^, with a regression equation of (R + G)/B = 2.82–0.04C (R^2^ = 0.9937) and a limit of detection (LOD) of 0.06 μg mL^−1^ (Fig. 4B) (LOD = 3σ/S, where σ is the standard deviation of the blank signal and S is the standard curve slope).

The TAC analysis could also be performed via fluorescence spectroscopy measurements. As shown in **Fig. S10**, the fluorescence emission intensity decreased gradually with the increase of quercetin concentration, and the concentration of quercetin and the fluorescence intensity (Y) exhibited a good linear relationship with C in the range of 50 to 2 × 10^4^ ng mL^−1^, with a linear equation of Y = 496.78–19.34C and an LOD of 3.1 ng mL^−1^ (Fig. 4C). One can see that this linear range was apparently wider than that with the smartphone colorimetric APP, along with a higher sensitivity.

In order to evaluate the selectivity of the TAC platform, several potential interferences (e.g., MgCl_2_, NaNO_2_, tryptophan, glucose, and bovine serum albumin (BSA)) were tested, and the results were evaluated via the quenching of the fluorescence emission intensity, ΔF = F_0_ − F, where F_0_ and F represent the fluorescence emission intensity of the blank group and the testing sample, respectively. As shown in Fig. 4D, effective quenching was observed with quercetin, but only negligible effects were observed with other interferences, confirming good selectivity of the FeNPs@NCNT based TAC platform.

The FeNPs@NCNT nanozymes also exhibited good stability. No significant change was observed with the fluorescence signal intensity even after repetitive tests for 5 consecutive weeks (Fig. 4E). The good stability of the FeNPs@NCNT nanocomposites was likely due to the strong interactions between the NCNT scaffold and loaded Fe NPs that helped anchor the nanoparticles and facilitated charge transfer across the interface.

### 3.6 TAC analysis of actual samples

**Fig. 5:**
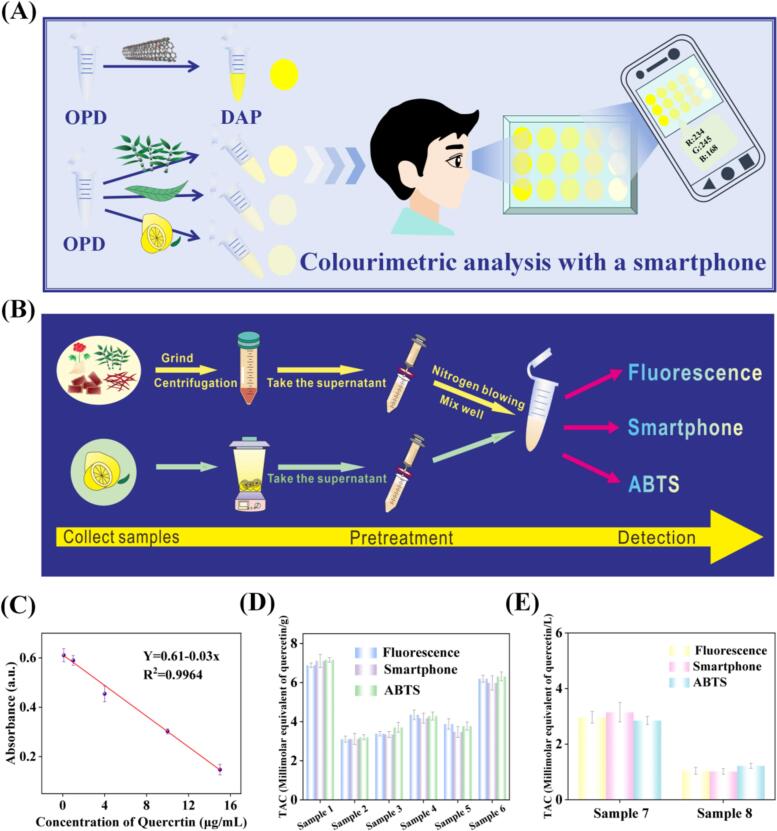
Practical applications of FeNPs@NCNT-based TAC analysis methodology. (A) Schematic diagram of the strategy for TAC analysis in real samples by naked-eye and smartphone. (B) Flowchart of the pre-treatment of real samples. (C) Linear relationship between quercetin concentration and absorbance (ABTS method). (D) Comparison of three different assays in detecting TAC in actual solid samples. (E) Comparison of three different assays in detecting TAC in actual liquid samples.

Notably, the FeNPs@NCNT based TAC platform showed a remarkable performance with actual samples, where the color change could be observed by naked eyes and quantitatively analysis with a smartphone (Fig. 5A) and fluorescence spectroscopy measurements (Fig. 5B). To validate the results, a comparative study was carried out using the standard method of TAC (ABTS method). Firstly, the linear range and linear equation of the ABTS method was investigated. As shown in Fig. 5C, the ABTS method showed a good linear relationship between the absorbance (A) and the quercetin concentration (C) within the range of 0.1–15 μg mL^−1^, A = 0.61–0.03C (R^2^ = 0.9964).

Subsequently, eight actual samples were pre-treated and detected using the FeNPs@NCNT-based TAC platform with the smartphone-based digital image colorimetric APP and fluorescence mode. The samples were also tested via the ABTS method. As shown in Fig. 5D-E, good agreement can be observed between the results obtained via the FeNPs@NCNT-based TAC analysis method and those from the ABTS method (**Table S2**), indicating that the FeNPs@NCNT-based TAC method is accurate and reliable, and can be used for real sample testing and analysis. In addition, the TAC of *Carthami flos* (Sample 1) and *Camellia sinensis* (Sample 6) were the highest among the eight real samples, suggesting that these two foods possessed the best antioxidant activity.

## 4 Conclusion

In this study, FeNPs@NCNT nanocomposites consisting of nitrogen-doped carbon nanotubes loaded with iron nanoparticles were successfully synthesized by high-temperature pyrolysis of MOF precursors. The FeNPs@NCNT sample exhibited excellent POD and OXD-like activity, as manifested in the colorimetric transformation of OPD to DAP, and could be exploited for the development of a dual-mode detection platform based on smartphone and fluorescence for TAC detection, using quercetin as the representative antioxidant which diminished the OPD to DAP color change. The smartphone-based strategy eliminated the need of complex instrumentation and was well suited for the rapid on-site analysis of TAC in resource-limited areas. The fluorescence detection mode showed a wide linear range (50–2 × 10^4^ ng mL^−1^) and high sensitivity (LOD = 3.1 ng mL^−1^). The FeNPs@NCNT-based sensing platform overcame the limitations of H_2_O_2_-dependent POD-like nanozymes and was successfully applied for TAC assessment of a range of actual samples. These results highlight the unique potential of nanozymes in the development of novel TAC assay tools and provide new ideas and directions for the future research and application of TAC assay technologies.

## CRediT authorship contribution statement

**Junlin Liu:** Writing – original draft, Investigation, Formal analysis, Data curation. **Sophia Xie:** Writing – original draft, Formal analysis, Data curation. **Nan Wang:** Formal analysis, Data curation. **Zhongyue Sun:** Formal analysis, Data curation. **Lina Tang:** Formal analysis, Data curation. **Guo-jun Zhang:** Project administration, Funding acquisition, Formal analysis. **John Tressel:** Formal analysis, Data curation. **Yulin Zhang:** Writing – original draft, Project administration, Funding acquisition, Formal analysis, Data curation, Conceptualization. **Yujie Sun:** Formal analysis, Data curation. **Shaowei Chen:** Writing – review & editing, Project administration, Funding acquisition, Formal analysis, Conceptualization.

## Declaration of competing interest

The authors declare that they have no known competing financial interests or personal relationships that could have appeared to influence the work reported in this paper.

## Data Availability

Data will be made available on request.

## Appendix A Supplementary data

Supplementary material Image 1
