# Supplementary material for: Iron nanoparticle/carbon nanotube composite as oxidase-like nanozyme for visual analysis of total antioxidant capacity
Source: Food Chem X. 2024 Dec 12;25:102093. doi: 10.1016/j.fochx.2024.102093 (PMC11721849; doi:10.1016/j.fochx.2024.102093)
Supplement: Supplementary file 1 — Supplementary material [file mmc1.pdf]

Supporting Information

Iron nanoparticle/carbon nanotube composite as oxidase-like nanozyme for visual analysis of total antioxidant capacity

Junlin Liu[a], Sophia Xie[b], Nan Wang[c]， Zhongyue Sun[a], Lina Tang[a], Guo-jun Zhang[a], John Tressel[d], Yulin Zhang[a,*], Yujie Sun[a,*] and Shaowei Chen[d,*]

[a] School of Laboratory Medicine, Hubei University of Chinese Medicine；Hubei Shizhen Laboratory, Wuhan 430065, China

[b] Wuhan Britain-China School, Wuhan, 430033, China

[c] Department of Physics, Jinan University, Guangzhou, 510632, China

[d] Department of Chemistry and Biochemistry, University of California, Santa Cruz, CA 95060, USA

* E-mail: zhangyulin2001@163.com.cn; fredsunsun@hotmail.com; shaowei@ucsc.edu.

List of Contents

- 10 figures
- 2 tables

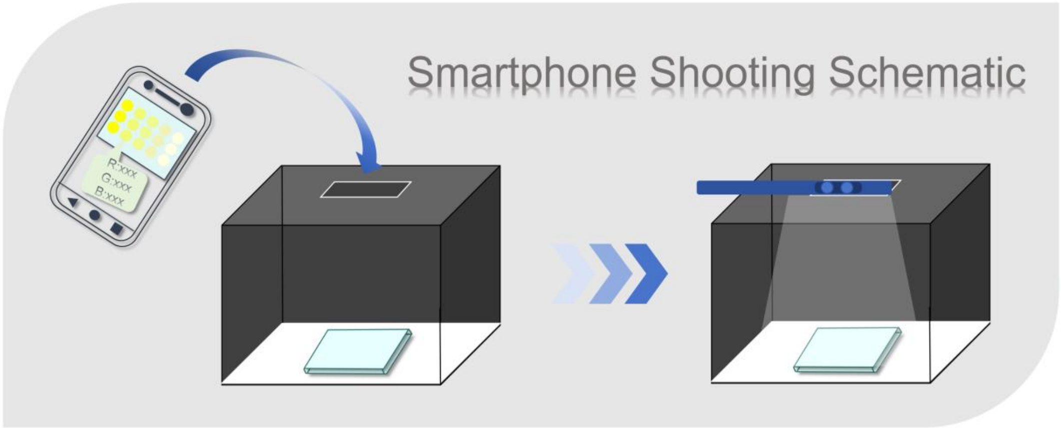

Figure S1. Smartphone shooting schematic.

The experimental procedure is detailed as follows: first, a 96-well plate containing the samples was placed in a homemade dark box, which was equipped with LEDs on the inner wall to provide uniform light intensity. In order to obtain unbiased spatial information, the rear camera of a smartphone was placed parallel to the 96-well plate. At the same time, a rectangular opening was designed on the top to allow the smartphone camera to take flicker-free digital images. Then, the red (R), green (G), and blue (B) values of the digital images were read using the "Color Picker" APP, and the TAC of the samples was evaluated by the linear relationship between the concentration of the antioxidant active ingredients and the RGB values.

Smartphone and smartphone camera parameters: The model of the tested phone is Mi 9 SE, Xiaomi Technology Co, China. Its main specifications include a 5.97-inch screen, 128 GB of RAM, an octa-core processor, and runs Android version 9.0. The rear camera used for the shooting process has 48 megapixels with a Sony IMX586 sensor and an aperture of f/1.75, which is capable of clearly capturing color variations in the reactive system.

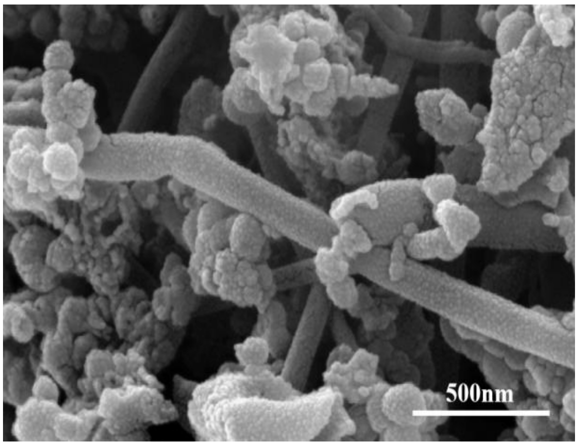

Figure S2. SEM image of FeNPs@NCNT.

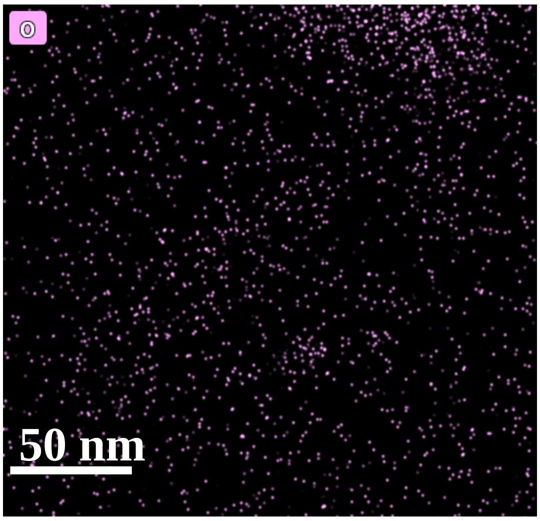

Figure S3. Elemental map of O in FeNPs@NCNT.

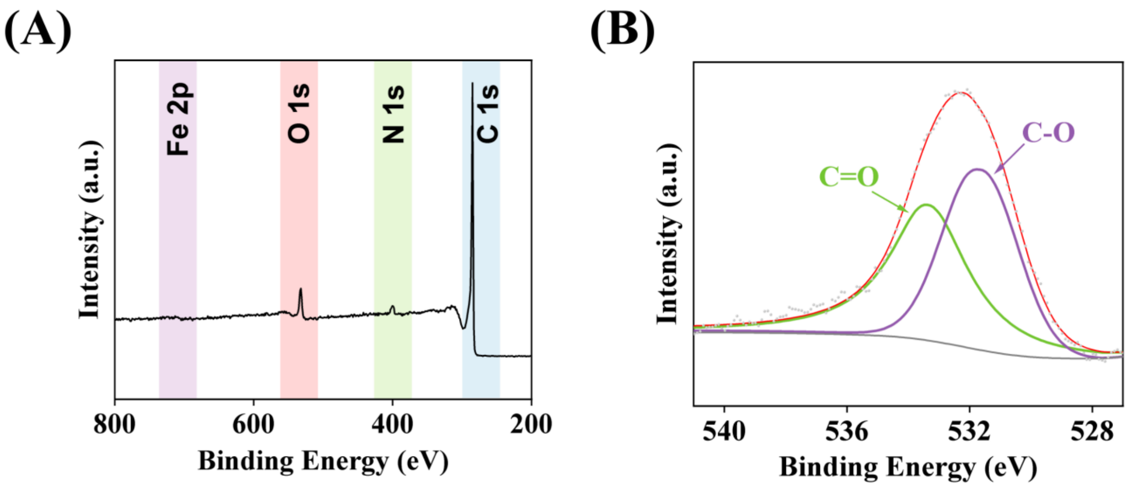

Figure S4. (A) XPS survey spectrum and high-resolution scan of the (B) O 1s electrons of FeNPs@NCNT.

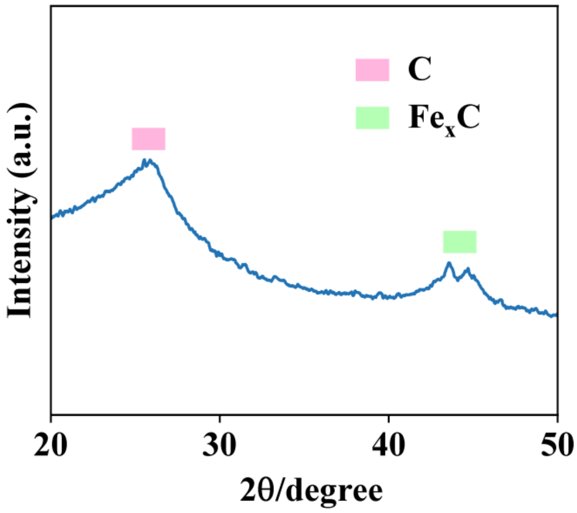

Figure S5. XRD patterns of FeNPs@NCNT.

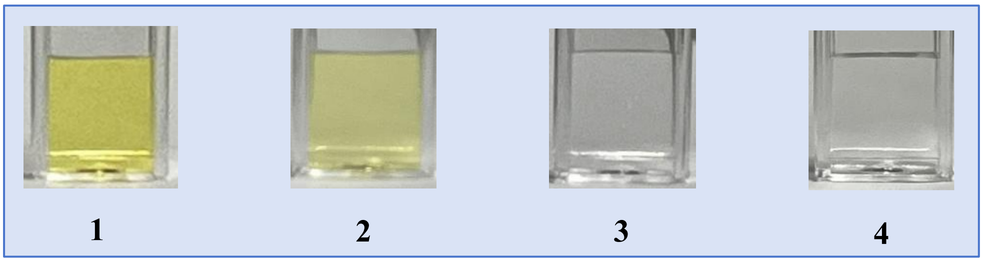

Figure S6. Photographs of various solutions after 30 min at room temperature: (1) FeNPs@NCNT+H_2O_2+OPD, (2) FeNPs@NCNT+OPD, (3) FeNPs@NCNT, and (4) OPD.

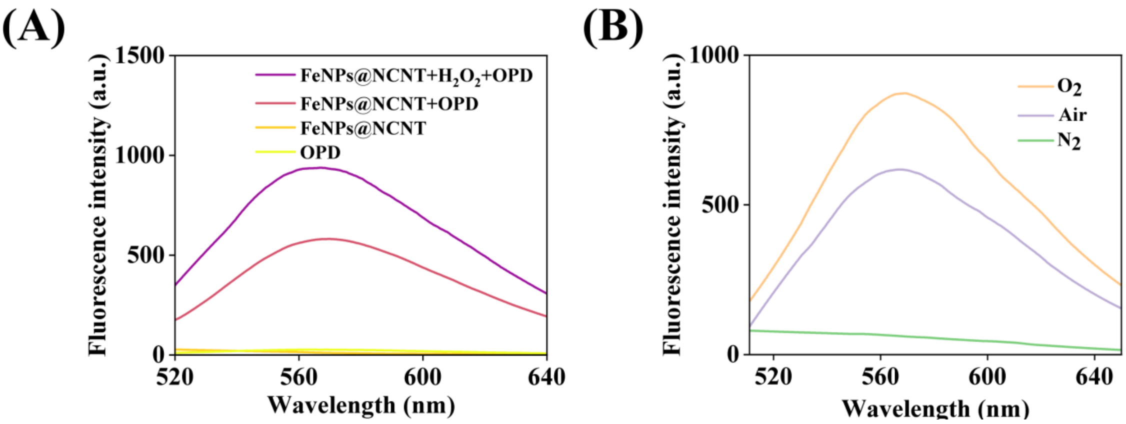

Figure S7. (A) Fluorescence emission spectra of various solutions. (B) Fluorescence emission spectra of the FeNPs@NCNT+OPD solution saturated with O_2, air or N_2. The excitation wavelength is all at 417 nm.

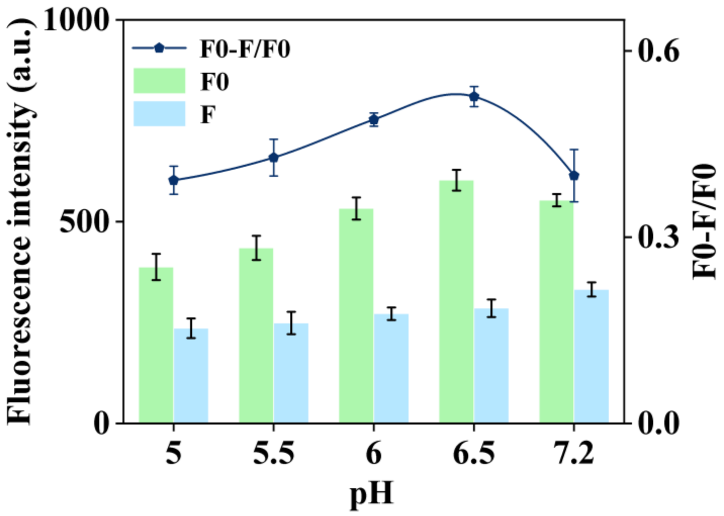

Figure S8. Effect of pH on the fluorescence emission intensity of .

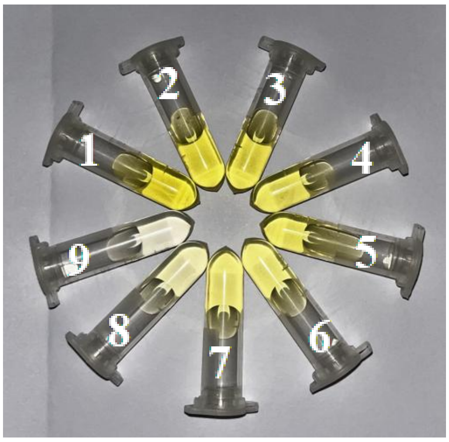

Figure S9. Photograph of the FeNPs@NCNT+H_2O_2 solution with the addition of quercetin at different concentrations: (1) 0, (2) 1×10, (3) 5×10, (4) 5×10^2, (5) 6×10^3, (6) 8×10^3, (7) 1×10^4, (8) 2×10^4, and (9) 3×10^4 ng mL^{-1}.

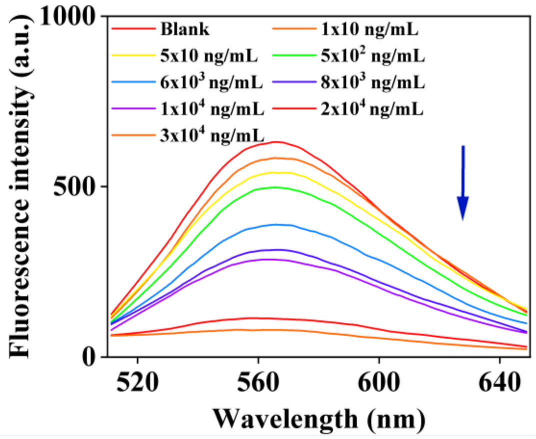

Figure S10. Fluorescence emission spectra of quercetin at different concentrations (excitation wavelength 417 nm).

Table S1. Compared the Kinetic parameters of various nanozymes.

Nanozyme	Substrate	Activity	K_m (mM)	V_{max} (M s^{-1})	Reference
Pd/g-C_3N_4	OPD	Oxidase	0.086	1.52 x 10^{-8}	(Zhang et al., 2022)
Au@Pt	OPD	Oxidase	0.268	1.53 x 10^{-8}	(Bonet-Aleta, Garcia-Peiro, Irusta, & Hueso, 2022)
$Ni_3V_2O_8$	OPD	Oxidase	12.43	1.43 x 10^{-8}	(Vetr, Fallah Moafi, & Moradi-Shoeili, 2023)
AgNPs@Fe_3O_4	OPD	Peroxidase	2.91	-	(Mazhani, Alula, & Murape, 2020)
AgNPs	OPD	Peroxidase	0.9133	-	(Alula, 2023)
FeNPs@NCNT	OPD	Oxidase	0.042	1.76 x 10^{-8}	This work

Table S2. Pearson's correlation coefficients.

Method		Pearson's
ABTS	Fluorescence	0.9960
ABTS	Smartphone	0.9938

References

Alula, M. T. (2023). Peroxidase-like activity of biosynthesized silver nanoparticles for colorimetric detection of cysteine. *Rsc Adv, 13*(24), 16396-16404. https://doi.org/10.1039/d3ra01587d.

Bonet-Aleta, J., Garcia-Peiro, J. I., Irusta, S., & Hueso, J. L. (2022). Gold-Platinum Nanoparticles with Core-Shell Configuration as Efficient Oxidase-like Nanosensors for Glutathione Detection. *Nanomaterials, 12*(5). https://doi.org/10.3390/nano12050755.

Mazhani, M., Alula, M. T., & Murape, D. (2020). Development of a cysteine sensor based on the peroxidase-like activity of AgNPs@Fe_3O_4 core-shell nanostructures. *Anal Chim Acta, 1107*, 193-202. https://doi.org/10.1016/j.aca.2020.02.021.

Vetr, F., Fallah Moafi, H., & Moradi-Shoeili, Z. (2023). Selective and sensitive colorimetric detection of Cr(VI) using porous $M_3V_2O_8$ (M= Ni, Cu, and Zn) nanostructures as oxidase mimics. *Int J Environ Anal Chem*, 1-23. https://doi.org/10.1080/03067319.2023.2223135.

Zhang, C. H., Ni, P. J., Wang, B., Liu, W. D., Jiang, Y. Y., Chen, C. X., . . . Lu, Y. Z. (2022). Enhanced oxidase-like activity of g-C_3N_4 nanosheets supported Pd nanosheets for ratiometric fluorescence detection of acetylcholinesterase activity and its inhibitor. *Chin Chem Lett, 33*(2), 757-761. https://doi.org/10.1016/j.cclet.2021.08.017.
